# The Molecular Characteristics of the *FAM13A* Gene and the Role of Transcription Factors *ACSL1* and *ASCL2* in Its Core Promoter Region

**DOI:** 10.3390/genes10120981

**Published:** 2019-11-28

**Authors:** Chengcheng Liang, Anning Li, Sayed Haidar Abbas Raza, Rajwali Khan, Xiaoyu Wang, Sihu Wang, Guohua Wang, Yu Zhang, Linsen Zan

**Affiliations:** 1 College of Animal Science and Technology, Northwest A&F University, Yangling 712100, Shaanxi, China; lcc20151120@nwafu.edu.cn (C.L.); lianning110.119@163.com (A.L.); haiderraza110@nwafu.edu.cn (S.H.A.R.); rajwalikhan@nwafu.edu.cn (R.K.); 2014050493@nwsuaf.edu.com (X.W.); sihumeng@126.com (S.W.); eywangguohua@126.com (G.W.); zhangyu147258@nwafu.edu.cn (Y.Z.); 2 National Beef Cattle Improvement Center, Northwest A&F University, Yangling 712100, Shaanxi, China

**Keywords:** *FAM13A*, *ACSL1*, *ASCL2*, promoter, carcass quality, preadipocytes

## Abstract

The gene family with sequence similarity 13 member A (*FAM13A*) has recently been identified as a marker gene in insulin sensitivity and lipolysis. In this study, we first analyzed the expression patterns of this gene in different tissues of adult cattle and then constructed a phylogenetic tree based on the FAM13A amino acid sequence. This showed that subcutaneous adipose tissue had the highest expression in all tissues except lung tissue. Then we summarized the gene structure. The promoter region sequence of the gene was successfully amplified, and the −241/+54 region has been identified as the core promoter region. The core promoter region was determined by the unidirectional deletion of the 5’ flanking promoter region of the *FAM13A* gene. Based on the bioinformatics analysis, we examined the dual luciferase activity of the vector constructed by the mutation site, and the transcription factors *ACSL1* and *ASCL2* were found as transcriptional regulators of *FAM13A*. Moreover, electrophoretic mobility shift assay (EMSA) further validated the regulatory role of *ACSL1* and *ASCL2* in the regulation of *FAM13A*. *ACSL1* and *ASCL2* were finally identified as activating transcription factors. Our results provide a basis for the function of the *FAM13A* gene in bovine adipocytes in order to improve the deposition of fat deposition in beef cattle muscle.

## 1. Introduction

With the improvement in living standards, high-end beef production has attracted more and more attention. The amount of intramuscular fat (IMF) in beef muscle tissue cross-sections can improve the palatability, juiciness, tenderness, and flavor of meat. The production of beef with good marbling properties can increase the profits of beef producers [1,2,3,4,5]. Therefore, it is important to explore the genes or pathways that control adipocyte differentiation. This process, also known as adipogenesis, is tightly regulated by a coordinated cascade of transcription factors interacting with each other to regulate the expression of downstream genes involved in adiposity and fat metabolism [6,7,8,9]. Transcription factors, e.g., PPARγ, are protein molecules that can bind to specific sequences upstream of the 5′ end of a gene, thus ensuring that target genes are expressed in a specific time and space. Transcription factors such as C/EBPα play a key role in adipocyte differentiation [10].

The full name of the *FAM13A* gene, namely, family with sequence similarity 13 member A, indicates its highly conservative sequence. Human *FAM13A* is highly expressed in adipose tissue, duodenum, placenta, and thyroid [11]. In previous studies conducted on human diseases [12,13,14], genome-wide association analysis shows that *FAM13A* is related to chronic obstructive pulmonary disease (COPD) [15]. COPD is a chronic lung disease characterized by incomplete irreversible airflow restriction and inflammation as well as lung parenchymal damage and airway reconstruction. In 2017, a study was conducted on the correlation between the *FAM13A* gene and susceptibility to COPD disease. In recent studies, DNA microarray analysis [16] was carried out in mice fed with high-adipocyte diet and in control mice. The top 20 genes with downregulated expression were identified, and included the *FAM13A* gene. 

The purpose of this study was to analyze the *FAM13A* gene and clone its promoter region through bioinformatics analysis and tissue expression. A 1000 bp (approx.) promoter region upstream of the 5’ end of the *FAM13A* gene was successfully cloned, with the core promoter region located at −241/+54 bp. The core promoter region was analyzed, and the key transcription factors were predicted; these transcription factors (TFs) were then verified using point mutation and electrophoretic mobility shift assay (EMSA). Two transcription factors, acyl-CoA synthetase long chain family member 1 (*ACSL1*) and Achaete-scute family bHLH transcription factor 2 (*ASCL2*), were identified as key activators in the core promoter region of *FAM13A* gene. These results provide a basis for functional research of the *FAM13A* gene in adipocytes of Qinchuan cattle.

## 2. Materials and Methods 

### 2.1. Ethics Statement

All animal handling was approved by Northwestern A&F University’s Experimental Animal Management Committee (EAMC). In accordance with the EAMC/20-23 statement on April 20, 2013, all institutions and government regulations were followed.

### 2.2. Construction of the Phylogenetic Tree of the FAM13A Gene

Amino acid homology comparison analysis was performed using the FAM13A gene sequence, mainly including the amino acid sequence of eight species: cattle (*Bos taurus*, accession number: NP_777117.1), Bison (*Bison bison*, accession number: XP_010858466.1), sheep (*Ovis aries musimon*, accession number: XP_012013830.1), goat (*Capra hircus*, accession number: XP_005681496.1), mice (*Mus musculus*, accession number: NP_705802.1), rat (*Rattus norvegicus*, accession number: NP_001094332.1), rhesus macaque (*Macaca mulatta*, accession number: XP_014994260.1), and human (*Homo sapiens*, accession number: NP_001015045.1). A phylogenetic tree of amino acid sequences was constructed using MEGA 7.0 software [17,18,19,20] (Philadelphia, PA, USA).

### 2.3. Tissue Expression Analysis of mRNA

The tissue samples were collected from the breeding farm of the National Beef Cattle Improvement Center of Northwest A&F University. The heart, liver, spleen, lung, kidney, subcutaneous fat, and visceral fat tissue samples from 18-month-old cows were aseptically collected from Qinchuan cattle. The tissue was cut into pieces and placed in a clean 50 ml centrifuge tube, quickly placed in liquid nitrogen, and then brought to the laboratory and stored in a refrigerator at −80 °C. Total RNA was extracted with TRIzol reagent (TakaraBio, Dalian, China), and the cDNA library was constructed according to the instructions of Prime Script RT Reagent Kit (TakaraBio, Dalian, China). Finally, a TB Green Premix Ex Taq II (Tli RNaseH Plus) (TakaraBio, Dalian, China) quantitative kit was used to prepare the mixture. The ABI 7500 system (Applied Biosystems) was used to carry out real-time fluorescence quantitative polymerase chain reaction (qPCR). The qPCR primer information is shown in Table 1, where 18S is the internal reference control primer, and the final raw data was analyzed by the 2^−ΔΔct^ calculation method [21].

### 2.4. FAM13A Gene Promoter Region Cloning

We used genomic DNA as a template to amplify the promoter region of the *FAM13A* gene by PrimeSTAR Max DNA Polymerase (TakaraBio, Dalian, China) amplification enzyme. The reaction conditions were 98 °C for 10 s, 55 °C for 5 s, 94 °C for 6 s, for 30 cycles. The different fragments were selected according to the prediction of NCBI, based on transcription factor binding sites in the promoter of the *FAM13A* gene (GenBank accession sequence: NC_03333.1). Primers were designed using computer software Primer Premier ver. 5.0 (PREMIER Biosoft, http://www.premierbiosoft.com/). Subsequently, 1% agarose gel electrophoresis was used to detect bands of the appropriate size, and were confirmed through sequencing (Sangon, China). In order to determine the core promoter region of *FAM13A* gene, five fragments (−898/+54, −659/+54, −512/+54, −241/+54, and −79/+54) were amplified through unidirectional deletion of the 5’UTR with specific primers containing enzyme sites of *Kpn*I and *Sma*I, respectively. After agarose electrophoresis and sequence confirmation, the PCR products were cloned into pGL3-Basic vector using a DNA ligation kit (TakaraBio, Dalian, China). DH5α competent cells were used for transformation, and after sequence confirmation, the plasmids were extracted using Endo-Free Plasmid Mini Kit (Omega Bio-tek, GA, USA). Finally, the recombinant vector was digested with two restriction enzymes.

### 2.5. Bovine Preadipocyte Culture and Cell Transfection

Bovine preadipocytes were collected from 4-day-old Qinchuan cattle at the National Beef Cattle Improvement Center of Northwest A&F University. The preadipocyte cells were maintained in growth media containing 90% F12/DMEM (Hyclone, New York, USA), 10% fetal bovine serum (PAN Biotech, Germany), and 1% antibiotics (100 IU/mL penicillin and 100 µg/mL streptomycin). The cells were plated in 24-well plates and grown under the influence of 70–90% cells at 37 °C and under 5% carbon dioxide. Cells were transfected using Lipofectamine 3000 (Invitrogen, CA, USA). The pRL-TK plasmid vector was used as an internal reference vector for standardizing transfection [22]. Cells were harvested 48 hours after transfection, and the Dual Luciferase Reporter Assay System (Promega, CA, USA) was used to determine the relative activity of dual luciferase [23,24,25].

### 2.6. Site-Specific Mutation of TFs and Mutation Vector Construction

The site-directed mutagenesis constructs were produced by the Fast Mutagenesis System (TransGene, Beijing, China). The potential transcription factor binding sites on the positive and negative chain of the *FAM13A* promoter were analyzed using the Genomatix (http://www.genomatix.de/) suite. The MatInspector program available online was used to ensure that the site-directed mutagenesis did not produce any new binding sites for TFs. We mutated the putative transcription factor binding sites for *ACSL1* and *ASCL2* with the corresponding primers (Table 2). 

### 2.7. EMSA Validation of Transcription Factor Binding

The bovine adipocytes were cultured in a 10 cm culture dish using DMEM/F12 complete medium, and the medium was discarded when the cell confluence reached about 90%.

Extraction of bovine precursor adipocyte total nucleoprotein was performed using Nuclear Extract Kit (Active Motif, CA, USA) kit followed by ultrasonic disruption of cells with Covaris M220, followed by centrifugation at 14,000× *g* for 10 min to collect supernatant, subpackaging with a 0.2 mL centrifuge tube without RNA enzyme in 30 μL, and storing in a refrigerator at −80 °C.

We chose a biotin-labeled probe, mutation probe, and competitive probe (Table 3). The sequence of biotin-labeled probe was identical with that of competitive probe, except that biotin was added at the 5’ end of the sequence. Biotin was easily bonded to the protein by a covalent bond. In this way, a stabilized streptavidin HRP avidin molecule in light shift chemistry kit (Thermo Fisher, Ma, USA) reacts with the biotin molecule binding to the specific protein, which not only plays a multilevel amplification role, but also makes the catalytic effect more obvious and easier to visualize. The transcription factor antibodies used in EMSA were *ACSL1* (Thermo Fisher, Invitrogen, AA8320N) and *ASCL2* (Abcam, ab157918).

The response system of each lane includes 1.0 μL 10× binding buffer, 0.5 μL 50% glycerol, and 0.5 μL 100 mM MgCl_2_ were added to each other lane. The complex of protein–deoxyribonucleic acid are released in 6% denatured polyacrylamide gel in 0.5× total alkaline buffer electrophoresis polyacrylamide run at 110 V for 75 minutes. A semi-dry film transfer imprinting system was used, with membrane transfer conditions of 200 mA 30 minutes, 5–10 cm with ultraviolet radiation, and 15 minutes of stitching. Light shift chemiluminescence kit (Thermo Fisher, MA, USA) was used for luminescence detection. Blocking with 15 mL Blocking Buffer for 15min, then adding 50 μL Stabilized Streptavidin-HRP for 15 min every 15 mL Blocking Buffer. Then wash with Wash Buffer (1×) for 4 times, each time for 5 minutes, and finally use the balancing solution Substrate Equilibration Buffer to balance for 5 minutes. Finally, in the production of luminescence and visualization using ChemDoc XRS System (BIO-RAD) using an appropriate amount of glowing liquids (Romanian solution/amplifier in configuration 1:1 used as a stable solution peroxide) [22,26,27].

### 2.8. Statistical Analysis

SPSS (version 16.0) was used to analyze the relative mRNA expression levels of the *FAM13A* gene in different bovine tissues of adult cattle, and the Duncan test was used to conduct multiple comparisons among groups. An independent-samples T test was used to analyze the relative luciferase activity of different promoters. All the data in the paper were expressed as mean ± standard deviation (SD). * *p* < 0.05, ** *p* < 0.01, *n* = 3

## 3. Results

### 3.1. Expression Patterns and Bioinformatics Analysis of FAM13A Genes 

**Figure 1:**
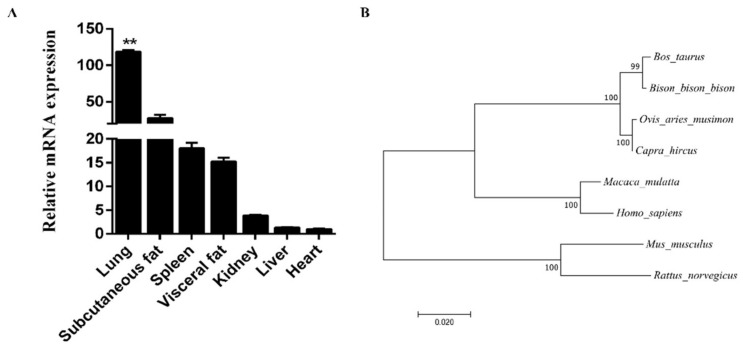
Expression patterns of *FAM13A* gene in various tissues of cattle and phylogenetic tree based on amino acid sequence. (**A**) Relative expression of *FAM13A* gene in seven major tissues of adult Qinchuan cattle (18 months old). (**B**) Phylogenetic tree of amino acids in *FAM13A* gene of 8 species including *Bos taurus*.

**Figure 2:**
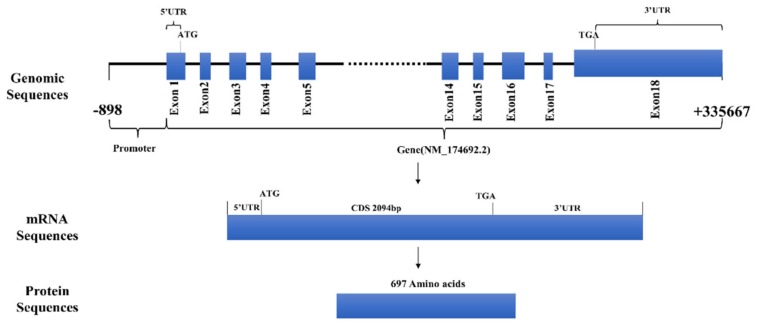
The gene structure of *FAM13A* including the genome level, mRNA level, and protein level.

We extracted total RNA from seven tissues and analyzed the tissue expression profiles. We found that *FAM13A* was expressed most in adipose tissues, except lung tissue (Figure 1A). We used MEGA 7.0 software to analyze the homologous amino acid sequences in eight species, including *Bos taurus*, bison, sheep, goat, mice, rat, rhesus monkey, and *Homo sapiens* [4]. The analysis found that *Bos taurus* had the highest homology with bison. The results are shown in Figure 1B. We summarized the gene structure of *FAM13A* and found that the gene is composed of 18 exons in the genome, and the length of the gene is approximately 57 kb. The NM_174692.2 mRNA transcript of the gene is 5125 bp in length and encodes 697 amino acids. The results are shown in Figure 2.

### 3.2. Transcription Factor Prediction and Enzyme Activity Determination of FAM13A Promoter Region.

**Figure 3:**
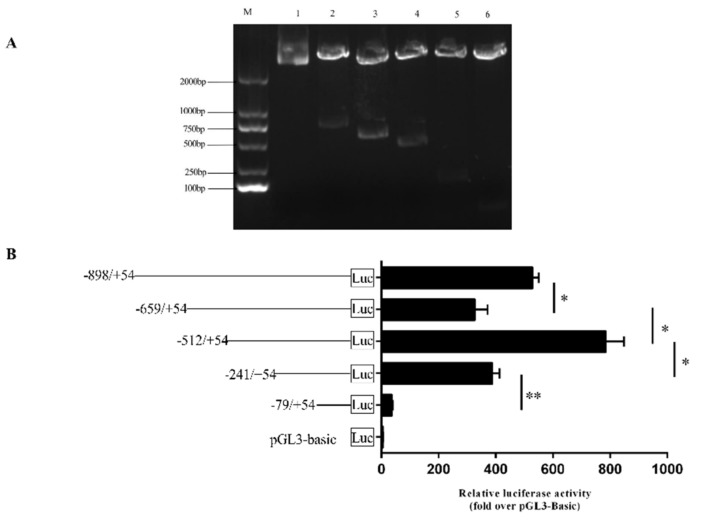
Construction of fragment-by-fragment deletion vector of *FAM13A* gene promoter and identification gel electrophoresis map of 5 recombinant vectors constructed by enzyme activity assay (**A**) −898/+54, −659/+54, −512/+54, −241+54, and −79/+54, where lane 1 is PGL3-basic empty vector and lanes 2–6 are different fragment-by-fragment deletions. (**B**) After transfecting recombinant PGL3-basic vector into bovine precursor adipocytes for 48 hours, double luciferase activity was measured and analyzed statistically (* indicates *P* < 0.05, ** indicates *P* < 0.01).

**Figure 4:**
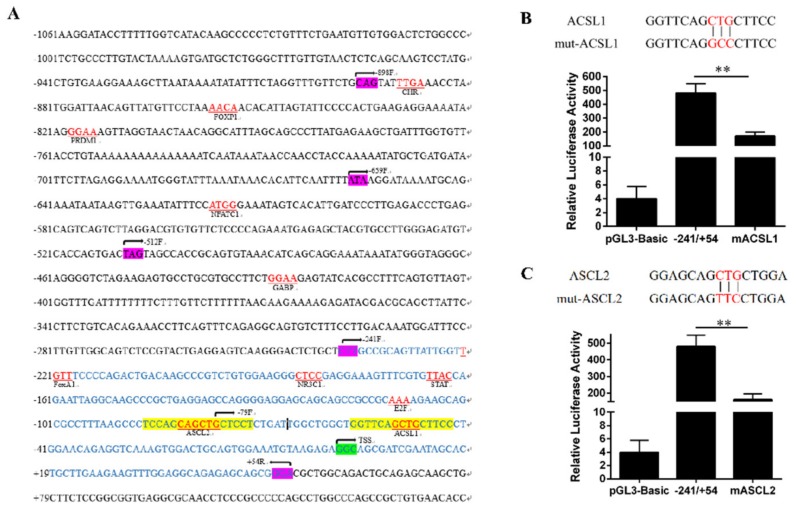
Results of transcription factor prediction and point mutation enzyme activity measurement in *FAM13A* promoter region. (**A**) Results of transcription factor prediction in promoter region (**B**) enzyme activity measurement of point mutation of transcription factor *ACSL1* (**C**) enzyme activity measurement of point mutation of transcription factor *ASCL2* (pGL 3-basic represents empty carrier group, −241/+54 represents core promoter region group); mACSL1 and mASCL2 represent the enzyme activity after mutation of *ACSL1* and *ASCL2* transcription factor binding site. ** means *P* < 0.01).

First, we constructed 5 recombinant vectors as shown in Figure 3A, then we conducted the luciferase activity assay for the identification of core promoter region of the *FAM13A* gene. The unidirectional deletion of the 5’UTR promoter region at −241/+54 fragment caused significant reduction in the luciferase activity (−79/+54). Therefore, we preliminarily identified −241/+54 bp as the core promoter region of *FAM13A* (Figure 3B). Then we predicted the transcription factors in the promoter region of *FAM13A* using the GenoMatix Online website. As shown by Figure 4A, the purple background marker sequence is the sequence position targeted by the fragment-by-segment primer. The blue marker sequence is the core promoter region sequence. Two transcription factors, *ACSL1* and *ASCL2*, were found in the core promoter region, wherein the yellow background marker sequence is the transcription factor *ACSL1* and *ASCL2* binding regions, and the red portion of the yellow background marker sequence is the transcription factor core binding site. *ASCL2* was screened out from the core promoter region. Next, mutation primers were designed according to the binding sites of these two key transcription factors, as shown in Figure 4B,C. The mutated vectors were transfected into bovine preadipocytes. The pGL3-basic empty vector was used as a control. The results of enzyme activity were analyzed. It can be seen that the enzyme activity of the mACSL1 and mASCL2 groups was significantly (*P* < 0.05) lower than the non-mutated group. These results indicate that the presence of *ACAL1* and *ASCL2* transcription factors can improve transcriptional activity of the *FAM13A* gene. 

### 3.3. EMSA Experimental Verification of ACSL1 and ASCL2 Transcription Factors

**Figure 5:**
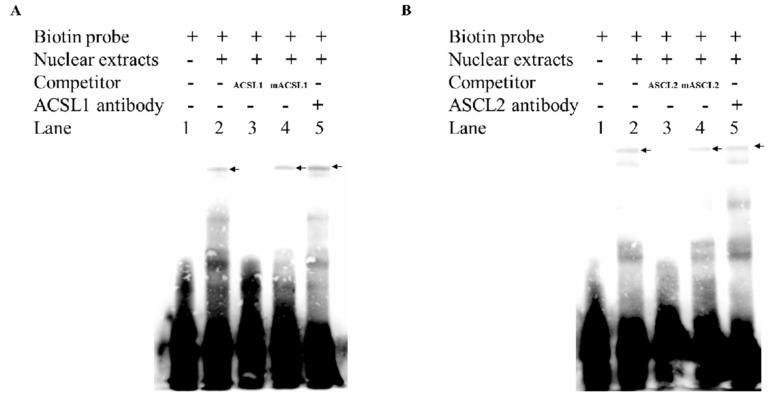
Electrophoretic mobility shift assay (EMSA) transcription factors *ACSL1* and *ASCL2*. (**A**) EMSA test verifies binding of transcription factor *ACSL1*. (**B**) EMSA test verifies binding of transcription factor *ASCL2*. Only biotin-labeled probes are added to lane 1, biotin-labeled probes and proteins are added to lane 2, labeled competitive probes are added to lane 3, mutation probes are added to lane 4, and antibodies corresponding to transcription factors are added to lane 5.

The results of electrophoretic mobility shift assay exhibited that the band of DNA–protein interaction could be significantly weakened or even eliminated after competitive probes were added, as in the third lane. After the antibody corresponding to the transcription factor was added to the fifth lane, the supershift band of Figure 5A was observed, but it was not obvious probably because the antibody formed a complex with the protein after the antibody was added, resulting in a molecular weight becoming large and, thus, the migration rate was slow. At the same time, it can be seen that the binding band in Figure 5B becomes shallow because some of the macromolecular complexes remain in the spotting holes due to electrophoresis time and current. This indicates that the *ACSL1* and *ASCL2* transcription factors do bind to the core promoter region of *FAM13A* and positively regulate the transcription of the *FAM13A* gene (Figure 5).

## 4. Discussion

The *FAM13A* gene family is comprised of three member genes including *FAM13A*, *B*, *FAM13C*, and *FAM13A* (also known as *PRECM1*), which are located on bovine chromosome 6, with 29 exons and 13 transcripts. Previous studies have shown [13] that this gene mainly acts on Rho GTPases and lung fibrosis signaling pathways, leading to the occurrence of COPD diseases [12,14]. However, in recent years, some researchers have found that *FAM13A* plays a role in mouse adipocytes and regulates adipocyte differentiation and lipolysis [10]. Moreover, the gene enhances the sensitivity of insulin in mouse cells, thereby ensuring systemic homeostasis [28,29,30,31].

In this study, we found that the *FAM13A* gene is highly conserved in livestock species, and *Bos taurus* sequence showed high similarity with bison species, which is consistent with systematic chemistry and the name source of the gene. Moreover, the expression level in lung tissue was significantly higher than in the other six tissues [32], thus conforming to the analysis of the gene as a candidate gene for human COPD diseases. However, we found high expression of *FAM13A* in subcutaneous adipose tissue after lungs, which shows its role in adipogenesis. This finding is in agreement with the results of the previous study [11] and indicates the significance of this research.

We also studied the function of *FAM13A* in adipocytes. It has already been established that *FAM13A* plays an important role in adipocyte proliferation and apoptosis. The regulatory effect on adipocyte differentiation is also being studied (the results have not yet been published). For the study of transcriptional regulation in the promoter region of *FAM13A*, we found *ACSL1* and *ASCL2* to act as transcriptional regulators of the *FAM13A* gene. Transcriptional regulation is a very important physiological process in all organisms. It is regulated by the synergistic action of transcription factors (TFs) and regulatory proteins, and plays an important role in the accuracy and diversity of the transmission of genetic information [25,33,34,35]. Transcription factor *ACSL1* belong to the long-chain adipocyte-acid CoA family, which can catalyze the conversion of long-chain adipocyte acids into acyl coenzyme A in active form, which is used to synthesize cell lipids and is degraded by β-oxidation [36,37,38,39,40]. In addition, PPARα participates in lipid metabolism and inflammatory reaction [41].

## 5. Conclusions

In summary, in the present study, we analyzed the gene structure of *FAM13A* and identified the core promoter region and two key transcription factors whose functions are mainly focused on adipocyte differentiation, lipid metabolism, and biological processes of cell proliferation and apoptosis. This study will provide a basis for future research to improve the meat quality of beef cattle.

## Figures and Tables

**Table 1:** The qPCR primer sequences and information of *FAM13A* gene.

Primer	Primer Sequence (5′–3′)	Annealing Temperature (°C)
18S-F	CCTGCGGCTTAATTTGACTC	57
18S-R	AACTAAGAACGGCCATGCAC	58
FAM13A-F	GTACCGCCTGGTCAAACAGATCCTA	64
FAM13A-R	TAGTTATCGTCTTCTGAACCCTC	57

**Table 2:** Primer information of segment-by-segment deletion of the promoter region of *FAM13A gene* and point mutation of key transcription factor binding sites.

Primer	Primer Sequence (5′–3′)	Annealing Temperature (°C)
FAM13A(−898)-F	CGGGGTACCCAGTATTTGAAACCTATGGATTAAC	55
FAM13A(−659)-F	CGGGGTACCATAAGGATAAAATGCAGAAATAATA	51
FAM13A(−512)-F	CGGGGTACCTAGTAGCCACCGCAGTGTAAACATC	62
FAM13A(−241)-F	CGGGGTACCCTTGCCGCAGTTATTGGTTGTTTCC	63
FAM13A(−79)-F	CGGGGTACCGCTCCTCTGATTGGCTGGGTGGTTC	67
FAM13A(+54)-R	TCCCCCGGGTCCCGCTGCTCTCTGCCTCCAAACT	69
mACSL1-F	GCTGGGTGGTTCAGACCCTTCCCTGGAA	71
mACSL1-R	GGTCTGAACCACCCAGCCAATCAGAGGA	70
mASCL2-F	TAAGCCCTCCAGCAGGCC CTCCTCTGAT	71
mASCL2-R	GGCCTGCTGGAGGGCTTA AAGGCGCTGC	74

**Table 3:** Biotin-labeled probe, competitive probe, and mutation probe information of key transcription factors in the *FAM13A* gene promoter region.

Primer	Primer Sequence (5′–3′)	Annealing Temperature (°C)
ACSL1-bio-F	Biotin-TGGCTGGGTGGTTCAGCTGCTTCCCTGGAACAGA	75
ACSL1-bio-R	Biotin-TCTGTTCCAGGGAAGCAGCTGAACCACCCAGCCA	75
ACSL1-jz-F	TGGCTGGGTGGTTCAGCTGCTTCCCTGGAACAGA	75
ACSL1-jz-R	TCTGTTCCAGGGAAGCAGCTGAACCACCCAGCCA	75
ACSL1-mut-F	TGGCTGGGTGGTTCAGACCCTTCCCTGGAACAGA	75
ASCL2-mut-R	TCTGTTCCAGGGAAGGGTCTGAACCACCCAGCCA	75
ASCL2-bio-F	Biotin-TAAGCCCTCCAGCAGCTGCTCCTCTGATTGGCT	74
ASCL2-bio-R	Biotin-AGCCAATCAGAGGAGCAGCTGCTGGAGGGCTTA	74
ASCL2-jz-F	TAAGCCCTCCAGCAGCTGCTCCTCTGATTGGCT	74
ASCL2-jz-R	AGCCAATCAGAGGAGCAGCTGCTGGAGGGCTTA	74
ASCL2-mut-F	TAAGCCCTCCAGCAGGCCCTCCTCTGATTGGCT	75
ASCL2-mut-R	AGCCAATCAGAGGAGGGCCTGCTGGAGGGCTTA	75

## Abbreviations

*FAM13A*: family with sequence similarity 13 member A; *ACSL1*: acyl-CoA synthetase long chain family member 1; *ASCL2*: Achaete-scute family bHLH transcription factor 2; EMSA: electrophoretic mobility shift assay; IMF: intramuscular fat; PPARγ: peroxisome proliferator activated receptor gamma; C/EBPα: CCAAT enhancer binding protein alpha; COPD: chronic obstructive pulmonary disease; EAMC: Experimental Animal Management Committee; FAM13B: family with sequence similarity 13 member B; FAM13C: family with sequence similarity 13 member C; TFs: transcription factors; PPARα: peroxisome proliferator activated receptor alpha.
